# A long-term follow-up study of adults with Chiari malformation type I combined with syringomyelia

**DOI:** 10.3389/fneur.2023.1274971

**Published:** 2023-12-01

**Authors:** Yan Hu, Mingchu Zhang, Chengcheng Duan, Dengpan Song, Mingkun Wei, Fuyou Guo

**Affiliations:** ^1^Department of Neurosurgery, The First Affiliated Hospital of Zhengzhou University, Zhengzhou, Henan, China; ^2^International Joint Laboratory of Nervous System Malformations of Henan Province, Zhengzhou, Henan, China

**Keywords:** Chiari malformation type I, syringomyelia, long-term, prognostic factors, cerebrospinal fluid (CSF)

## Abstract

**Background:**

There is a considerable amount of controversy regarding the treatment and prognosis of adult patients with Chiari malformation type I (CM-I) at home and abroad; furthermore, no large-sample, long-term, follow-up studies have examined CM-I patients with syringomyelia (SM) comparing posterior fossa decompression with resection of tonsils (PFDRT) vs. posterior fossa decompression with duraplasty (PFDD).

**Objective:**

This study retrospectively analyzed the factors affecting the treatment and long-term prognosis of adults with CM-I combined with SM.

**Methods:**

We retrospectively analyzed data from 158 adult CM-I patients combined with SM who underwent PFDRT or PFDD, including 68 patients in group PFDRT and 90 patients in group PFDD. We examined the clinical manifestations, imaging features, and follow-up data of patients. Clinical outcomes were assessed using the Chicago Chiari Outcomes Scale (CCOS), and radiographic outcomes were indicated by the syrinx remission rate. Multivariate logistic regression analysis and multiple linear regression analysis were used to explore the relevant factors affecting the long-term prognosis of patients.

**Results:**

This study showed that compared with preoperative patients in the PFDRT group and PFDD group, the sensory impairment, cough-related headache, and movement disorder were significantly improved (*p* < 0.01); meanwhile, the diameter of the syrinx and the volume of the syrinx decreased significantly (*p* < 0.001). Additionally, the study found that there were significant differences in the syrinx remission rate (*p* = 0.032) and the clinical cure rates (*p* = 0.003) between the two groups. Multivariate logistic regression analysis showed that age (*p* = 0.021), cerebellar-related symptoms (*p* = 0.044), preoperative cisterna magna volume (*p* = 0.043), and peak systolic velocity (*p* = 0.036) were independent factors for clinical outcomes. Multiple linear regression analysis showed that different surgical procedures were positively correlated with the syrinx remission rate (*p* = 0.014), while preoperative syrinx diameter (*p* = 0.018) and age (*p* = 0.002) were negatively correlated with the syrinx remission rate.

**Conclusion:**

In conclusion, this study suggested that, in a long-term follow-up, although both surgical procedures are effective in treating patients with CM-I and SM, PFDRT is better than PFDD; age and cerebellar-related signs independently affect the patient’s prognosis. Additionally, an effective prognosis evaluation index can be developed for patients, which is based on imaging characteristics, such as preoperative cisterna magna volume, preoperative syrinx diameter, and preoperative cerebrospinal fluid (CSF) hydrodynamic parameters to guide clinical work.

## 1 Introduction

Chiari malformation (CM) is a common congenital developmental disorder of the nervous system (1). In 1891, Hans Chiari was the first to describe hydrocephalus and congenital anomalies affecting the cerebellum (2). Due to advances in research, scholars at home and abroad currently believe that the CM should be divided into eight subtypes: type 0, type I, type 1.5, type II, type III, type 3.5, type IV, and type V (2–5). Among them, Chiari malformation type I (CM-I) is one of the most common clinical subtypes (6). Bone structure and brain tissue structure abnormalities cause cerebellar tonsil hernia to 5 mm below the foramen magnum plane (the hernia can even extend into the spinal canal), often accompanied by syringomyelia (SM) and neurological abnormalities (7). Some studies showed that symptomatic SM prevalence is 3.01:100,000 and incidence is 0.51:100,000; meanwhile, asymptomatic SM prevalence is 1.83:100,000 and incidence is 0.31:100,000. In addition, the symptomatic CM prevalence is 3.10:100,000 and incidence is 1.23:100,000; the asymptomatic CM prevalence is 4.64:100,000 and incidence is 1.85:100,000 (8–10). Studies of patients undergoing surgery have reported a prevalence of SM from 60 to 85% of patients with CM-I (11, 12). Patients with CM-I and SM have clinical symptoms, most of which are progressive aggravating trends; furthermore, some patients in the late stage may exhibit serious irreversible neurological dysfunctions (e.g., “Charcot’s Joint,” “claw hand,” limb paralysis, and incontinence), while some patients with medulla and brain stem compression may cause acute death (13). Therefore, clinical researchers have focused on how to effectively diagnose and treat patients with CM-I and SM. In patients with definite symptoms of CM-I, surgical procedures are the only recommended treatment. At present, there are many surgical methods that vary in efficacy, including simple posterior fossa decompression (PFD), posterior fossa decompression with duraplasty (PFDD), and posterior fossa decompression combined with the resection of tonsils (PFDRT). It remains unclear how to preoperatively predict the efficacy of surgery and how to predict the long-term effects with objective indicators. In recent years, phase-contrast magnetic resonance imaging (PC-MRI), as a new non-invasive examination in the evaluation of cerebrospinal fluid (CSF) flow, has promoted the pathophysiology of CSF circulation research progress, and CSF hydrodynamic parameters have been increasingly recognized as a predictive factor for the surgical effect and prognosis (14–20). Therefore, based on the current status of the diagnosis and treatment of CM-I combined with SM, this study retrospectively analyzed 158 patients with symptomatic CM-I combined with SM who presented to our hospital and underwent PFDRT or PFDD from January 2013 to June 2020. The present study evaluates the long-term outcome of surgical management of adult CM-I combined with SM according to clinical features and magnetic resonance imaging (MRI) findings, stressing some factors which might affect the outcome and accordingly may help guide surgical decision-making.

## 2 Materials and methods

### 2.1 Patient information

In accordance with the latest international expert consensus on the diagnosis and treatment of CM-I (21), patients who were admitted to the neurosurgery department of our hospital from January 2013 to June 2020 were screened. A total of 535 patients were diagnosed with CM-I, including 472 adult patients, and 359 patients also had SM. Based on the inclusion criteria, 237 study subjects were determined to be eligible. Of these, 79 patients were excluded from the study due to incomplete clinical data or loss of follow-up. Ultimately, a total of 158 adult patients with CM-I and SM were included in this retrospective study, including 68 patients in the PFDRT group and 90 patients in the PFDD group. All patients were treated by two experienced surgeons from our institution, and the choice of surgical operation was decided after communication with the patient because some patients refused to undergo cerebellar tonsil resection. This study has obtained informed consent from family members and patients.

### 2.2 Inclusion criteria

The inclusion criteria were as follows: (1) age > 18 years old; (2) after admission, patients underwent complete imaging examination and to confirm the diagnosis of CM-I (i.e., the cerebellar subtonsillar hernia was ≥5 mm under the foramen occipital plane), including 64 rows of the craniocervical junction (computed tomography, CT), over-flexion-extension X-ray photographic examination, 3.0 T craniocervical junction MRI, and foramen magnum region CSF flow imaging examination; (3) patients had CM-I combined with SM and showed clinical symptoms; (4) patients only received surgical treatment with either PFDRT or PFDD; (5) all patients were followed up for more than 3 years and provided complete follow-up data.

### 2.3 Exclusion criteria

The exclusion criteria were as follows: (1) secondary SM caused by spinal cord tumor, spinal cord injury, or spinal cord; (2) other diseases caused subtonsillar hernia, such as hydrocephalus, idiopathic increased intracranial pressure (ICH), or cerebellar tumor; (3) non-surgical treatment for CM-I combined with SM; (4) history of posterior fossa surgery; (5) unstable cranial and cervical connection; (6) other types of CM with or without SM; (7) family members refused to complete follow-up examinations.

### 2.4 Study methods

We collected patients’ basic information, including age, sex, blood pressure, disease duration (pre-operative symptom duration), operation time, hospital stay, and imaging characteristics. The clinical symptoms of patients can be divided into the following categories: (1) paraesthesia, such as numbness and separable sensory disorders in limbs or bodies; (2) cough-related headache, such as cough and valsalva action aggravated headache; (3) non-cough pain, such as headache, neck, and occipital pain (we used the Simplified McGill Pain Questionnaire (SF-MPQ) to assess patients’ pain); (4) motor dysfunction, such as limb weakness and muscle atrophy; (5) posterior neurological signs, such as dysphagia, hoarseness, and nystagmus; (6) cerebellar-related signs, such as ataxia and gait instability; (7) cervical bulbar compression signs, such as increasing in muscle tone, hyperreflexia, and bladder sphincter disorders.

**Figure 1:**
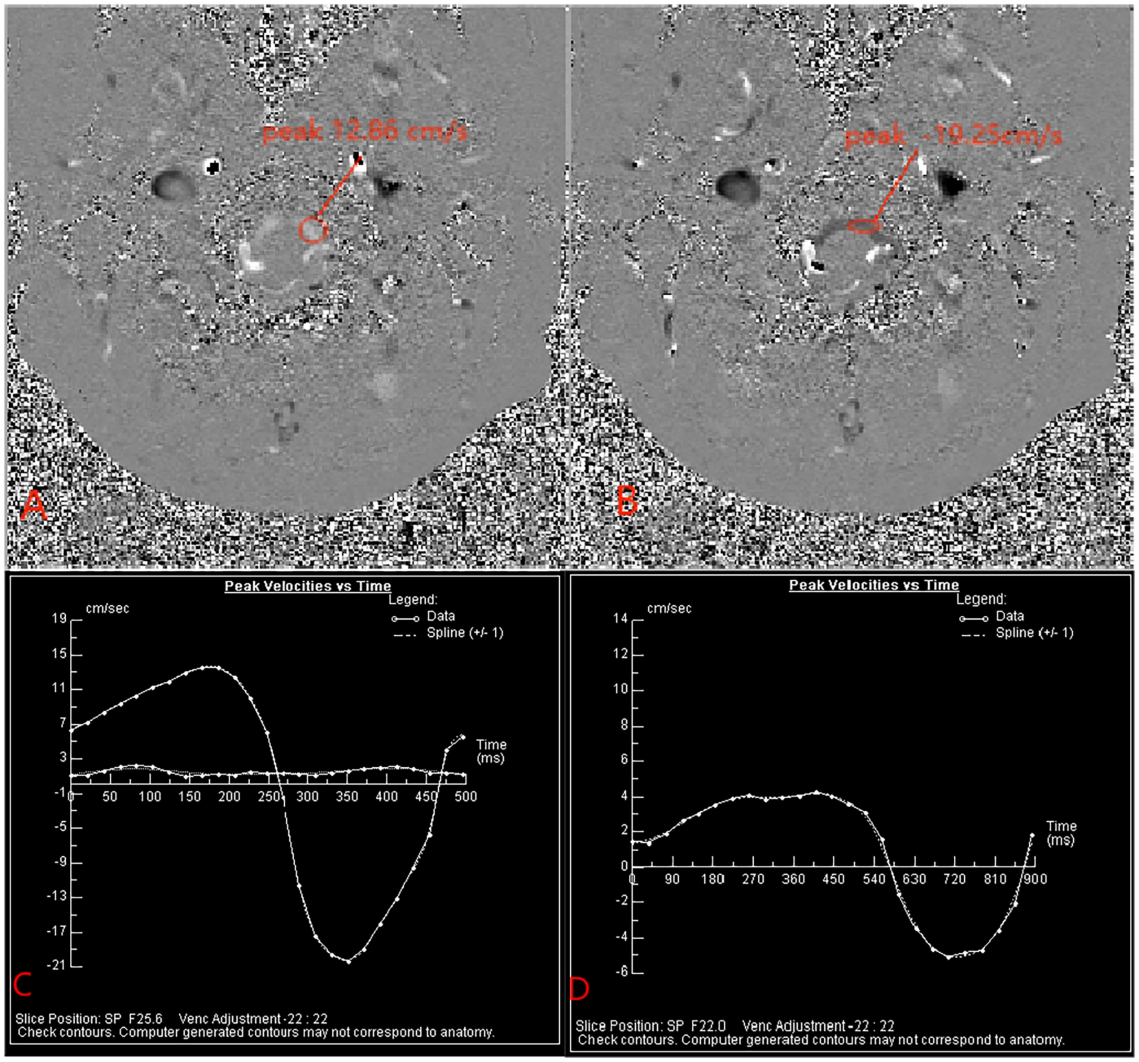
Panels **(A,B)** show the peak velocity of CSF during systole and diastole, respectively, before surgery. Panel **(C)** is the peak velocity time change curve in the patient’s preoperative cardiac cycle. Panel **(D)** is the peak velocity time change curve of the patient’s postoperative cardiac cycle.

We selected the median sagittal position to measure the length of the cerebellar subtonsillar hernia before and after surgery, and we selected the axial position to measure the preoperative syrinx maximum anterior and posterior diameter (defined as a) as well as the preoperative spinal cord diameter in the same plane was defined as b. The postoperative measurement of syrinx diameter was defined as a1, the postoperative spinal cord diameter in the same plane was defined as b1, the preoperative Vaquero index (VI), the ratio of the greatest diameter of the syrinx to that of the spinal cord was defined as a/b, and the postoperative VI was defined as a1/b1. Additionally, a Siemens superconducting 3.0 T Verio magnetic resonance instrument was used in imaging. Patients were placed in the supine position, with an 8-channel phased array cranial coil and ECG gating technology. PC-MRI sequence scanning was performed on the transverse section of the patient’s craniocervical junction. We define the flow of CSF in the cranial direction as positive (+) and the caudal direction as negative (−). At the post-processing workstation, a neuroradiologist measured the systolic CSF flow velocity, diastolic CSF flow velocity, and net flow at this level. The scan parameters were as follows: TR/TE of 25/8 ms (minor variations in TR according to heart rate), flip angle of 10°, matrix of 205 × 256, field of view of 15 × 15 cm, slice thickness of 5 mm, and velocity encoding of 22 cm/s (Figure 1).

Based on the 3D reconstruction technology of the open-source software 3Dslicer5.2.1, the syrinx volume and cisterna magna volume of patients were measured. Before the measurement began, we determined the specific range of each measurement index based on previous studies. The fourth ventricles are similar to a tent and are located between the medulla, pons, and cerebellum. They are connected by the main pipe of the midbrain and the lower spinal cord, with a clear anatomical structure and easy measurement from the specific imaging results of the patients. When the syrinx diameter is <1 mm, the SM returns to normal, and the medullary pool is the spatial structure of the cerebellum and occipital area. In the present study, the cisterna magna was understood as the space occupied by the CSF in the foramen occipital region, which is the space of all subarachnoid spaces involved in the CSF circulation within this region. Therefore, we define the upper boundary of the occipital pool as the plane at the level of the bridge and the inner tuberosity; we define the lower boundary as the plane through the lower margin of the atlas; and we define the occipital pool volume as the space occupied by the skull cavity and the CSF in the spinal canal between the two planes.

### 2.5 Surgical procedures

#### 2.5.1 PFDD

**Figure 2:**
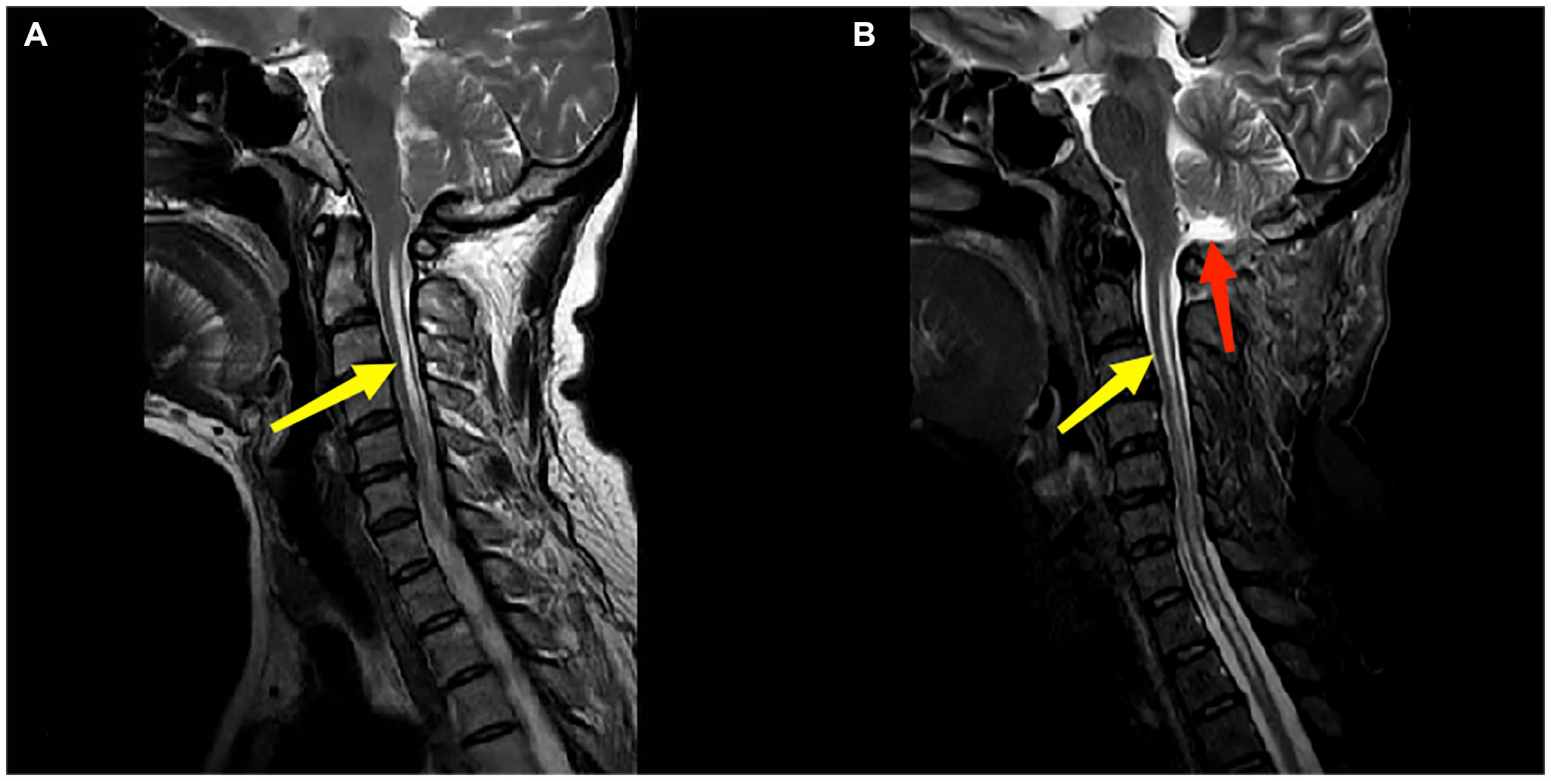
Panels **(A,B)** are the preoperation and postoperation MRI images of a 51-year-old female patient who underwent PFDD treatment. The red arrow in **(B)** indicates the postoperative recreated cistern magna. The yellow arrow indicates the patient’s syringomyelia, and there was a reduction in the diameter of syrinx postoperatively.

After general anesthesia, the neck was slightly bent forward, and the head was fixed with a three-nail head frame, and the posterior median incision was extended from the top of the top to the second cervical spine. The skin and the posterior arch were cut, thus forming a bone window of approximately 3*3 cm^2^. Hyperplastic and/or calcified atlantooccipital fascia were removed to achieve adequate bony decompression. “Y” type cuts were applied to the dura mater with the aim of keeping the arachnoid membrane intact and minimizing bloody contamination of the subarachnoid space. When a large arachnoid occipital area scar formation, sharp separation scar, or adhesion was observed, the CSF cycle was fully performed. The surgical area was flushed with hormone water, thereby reducing bloody stimulation and subarachnoid adhesion, and a non-absorptive suture was used for the autologous fascia, thereby reducing the rejection caused by artificial dural and arachnoid inflammation risk. With the cooperation of the anesthesiologist, the thoracic pressure of the patient was increased, the effect of a watertight suture was evaluated by mimicking Valsalva movements, and finally, the incision was closed layer by layer at the anatomical level (Figure 2).

#### 2.5.2 PFDRT

**Figure 3:**
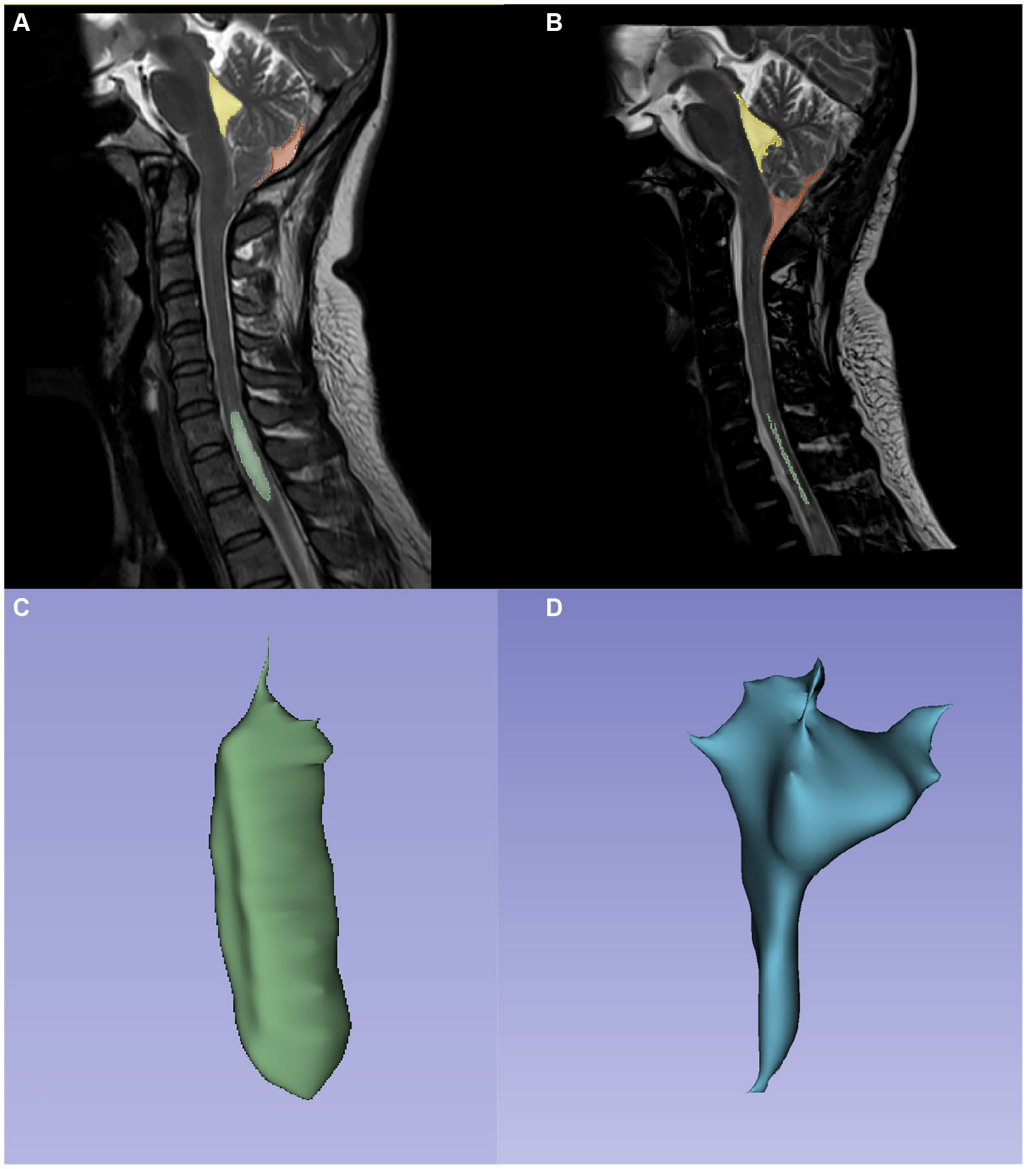
Panels **(A,B)** are the preoperation and postoperation MRI images of a 60-year-old male patient who underwent PFDRT treatment. Both in **(A,B)**, yellow, fraction and green represent the fourth ventricle, cistern magna, and SM area, respectively. Panels **(C,D)** are the SM and cisterna magna models drawn by 3Dslicer, respectively.

The same method was used to achieve bone decompression, and then a “Y” type cut was applied to the dura mater to sharply remove the arachnoid adhesion on the surface of the cerebellar tonsil, between the intercerebellar tonsils, and below the cerebellar tonsil. An absorbable gelatin sponge was placed behind the cerebellar tonsils to protect the brain stem. Bipolar low-frequency electrocoagulation was applied from the middle of the cerebellar tonsils to the two sides. During the operation, a surgeon was devoted to the protection of the posterior cerebellar artery to avoid vascular injury as much as possible. If the patient’s cerebellar subtonsillar hernia location was low or if fibrous hyperplasia was obvious, subsoft membrane resection was performed until the cerebellar tonsils were retracted to the level of the superior margin of the foramen magnum. We searched for the latches of the fourth ventricles; if any latches were closed, we excised them to fully open through the CSF circulation. After ensuring complete intracranial hemostasis, the same method was applied, and the incision was closed (Figure 3).

### 2.6 Patient evaluation and follow-up

Physicians evaluated patients based on their status at follow-up and score patients based on the Chicago Chiari Outcome Scale (CCOS), which are the clinical outcomes for each patient. The rating scale included four categories: pain symptoms, non-painful symptoms, ability to perform routine duties, and surgical complications. The sum of these four categories produces a composite score ranging from 4 to 16 points, reflecting the overall outcome of patient recovery. Scores of 4–8 indicate poor recovery, scores of 9–12 indicate stable recovery, and scores of 13–16 indicate good recovery. In this study, good recovery was defined as cured, while stable and poor recovery was defined as not cured. The syrinx remission rate was used as the basis of imaging results, which was defined as the ratio of the difference between the preoperative and postoperative syrinx volumes and the preoperative syrinx volume, which accurately reflects the syrinx response by three-dimensional volume measurement.

### 2.7 Statistical analysis

Data analysis was performed by using SPSS 26.0. The normality of the sample distribution was assessed. Normally distributed quantitative data are expressed as the mean ± standard deviation ( $\bar{x}\pm s$ ), non-parametric data were expressed as medians and compared using McNemar’s test, while categorical data are expressed as frequencies and percentages [*n* (%)]. Differences in clinical symptoms and imaging results between groups were examined using the independent sample *t*-test, chi-square test, or continuous correction chi-square test based on the normality of the distribution. The paired *t*-test was used to compare these variables before and after surgery. Univariate and multivariate logistic regression analyses were used to evaluate the factors affecting patients’ clinical outcomes. Bivariate correlation analysis and multiple linear regression analysis were used to identify preoperative variables related to the SM remission rate. To comprehensively evaluate the relationship between independent variables and study subjects, factors that were significant at a *p*-value of <0.10 in the univariate logistic regression analysis and correlation analysis were subsequently included in the multivariate logistic regression analysis and multiple linear regression analysis, respectively. A *p*-value of <0.05 was considered to indicate statistical significance.

## 3 Results

### 3.1 Basic preoperative information

A total of 158 subjects were included in the study, including the PFDRT group (*n* = 68) and PFDD group (*n* = 90). In the PFDRT group and PFDD group, the ratio of males to females was 1:3.25 vs. 1:2.21 (*p* = 0.292), respectively; the mean age was 47.97 ± 10.83 years vs. 46.14 ± 10.75 years (*p* = 0.293), respectively; and the mean duration was 12 [6, 114] months vs. 33 [6, 96] months (*p* = 0.376), respectively. No significant differences in operation time, hospital stay, and blood pressure were observed (*p* > 0.05); the details are shown in Table 1.

**Table 1:** Demographic before operation.

Feature	PFDRT (*n* = 68)	PFDD (*n* = 90)	*P*
Sex			0.292
Male	16 (23.5%)	28 (31.1%)	
Female	52 (76.5%)	62 (68.9%)	
Age (years)	47.97 ± 10.83	46.14 ± 10.75	0.293
Duration of symptoms (months)	12 [6,114]	33 [6,96]	0.376
Operation time (hours)	3.09 ± 0.97	3.15 ± 0.93	0.67
Time of hospital stay (days)	19.57 ± 7.18	19.36 ± 7.31	0.852
Blood pressure			0.294
No hypertension	43 (63.2%)	64 (71.1%)	
Hypertension	25 (36.8%)	26 (28.9%)	
**Follow-up time (months)**			
Mean	73 [47, 95]	67 [51, 87]	0.664
Range	[32, 119]	[31, 117]	

### 3.2 Clinical and imaging results

The results revealed that patients in both groups showed significant improvements in sensory disturbance, cough-related headache, and movement disorders (*p* < 0.01) than preoperative patients; the cervical bulbar compression signs and the posterior neurological signs improved by 100%. Meanwhile, the signs of non-cough-related headache and cerebellar-related signs did not improve significantly after the surgery (*p* > 0.05). A comparison between two groups of patients showed that the prognosis of cough-related headache in the PFDRT group was significantly better than that in the PFDD group (*p* = 0.036), and there was no significant difference in the prognosis of other signs (*p* > 0.05).

The present study showed that the maximum diameter of syrinx VI, the length of cerebellar tonsillar herniation, and the volume of syrinx were significantly reduced after operation in patients in both groups compared with those before operation (*p* < 0.001). Patients in both groups showed significant increases in the volume of the cisterna magna (*p* < 0.001). No significant changes in the volume of the fourth ventricles were found (*p* > 0.05). Patients in the PFDRT group showed a better cisterna magna volume (*p* < 0.001), syrinx remission rate (*p* = 0.044), and normal rate of cerebellar tonsils (*p* = 0.006) than patients in the PFDD group. Due to analysis, PC-MRI results showed that there was no significant difference in CSF dynamics parameters between the two groups (*p* > 0.05). Paired t-tests revealed that the CSF dynamics parameters improved after surgery, with the systolic peak and diastolic peak velocity decreasing (*p* < 0.001) and the net CSF flow increasing (*p* < 0.001; Tables 2–4).

**Table 2:** Basic clinical symptoms of patients.

Feature	PFDRT (*n* = 68)	PFDD (*n* = 90)	*P*
**Paraesthesia [*n*(%)]**			
Preoperation	45 (66.2%)	58 (56.7%)	0.225
Postoperation	6 (8.8%)	12 (24.1%)	0.377
*P*	<0.001^*^	<0.001^*^	
**Cough-related headache [*n*(%)]**			
Preoperation	22 (32.4%)	28 (31.1%)	0.868
Postoperation	2 (2.9%)	11 (12.2%)	0.036^*^
*P*	<0.001^*^	0.002^*^	
**Non-cough headache [*n*(%)]**			
Preoperation	7 (10.3%)	5 (5.6%)	0.266
Postoperation	2 (2.9%)	2 (2.2%)	1
*P*	0.168	0.441	
**Motor dysfunction [*n*(%)]**			
Preoperation	17 (25.0%)	29 (32.2%)	0.322
Postoperation	4 (5.9%)	8 (8.8%)	0.48
*P*	<0.001^*^	<0.001^*^	
**Cervical bulbar compression [*n*(%)]**			
Preoperation	2 (2.9%)	3 (3.3%)	
Postoperation	0	0	1
*P*	-	-	-
**Cerebellar-related signs [*n*(%)]**			
Preoperation	5 (7.4%)	5 (5.6%)	0.897
Postoperation	1 (1.5%)	3 (3.3%)	0.821
*P*	0.21	0.718	
**Posterior neurological signs [*n*(%)]**			
Preoperation	2 (2.9%)	4 (4.4%)	0.945
Postoperation	0	0	
*P*	-	-	

^*^p < 0.05.

**Table 3:** Clinicoradiological characteristics.

Feature	PFDRT (*n* = 68)	PFDD (*n* = 90)	*P*
**Maximum diameter of syrinx (mm)**			
Preoperation	5.09 ± 2.68	5.22 ± 2.52	0.749
Postoperation	2.89 ± 2.15	2.77 ± 2.00	0.701
*P*	*<*0.001^*^	*<*0.001^*^	
**VI**			
Preoperation	56.44% ± 21.44%	55.46% ± 20.93%	0.772
Postoperation	34.19% ± 22.17%	36.48% ± 20.39%	0.502
*P*	*<*0.001^*^	*<*0.001^*^	
**Tonsil descent (mm)**			
Preoperation	8.58 ± 3.73	9.22 ± 4.59	0.351
Postoperation	1.66 ± 2.82	3.29 ± 3.78	0.003*
*P*	*<*0.001^*^	*<*0.001^*^	
**Volume of syrinx (cm**^**3**^**)**			
Preoperation	4.06 ± 2.98	4.56 ± 4.24	0.35
Postoperation	1.46 ± 1.85	1.58 ± 2.01	0.702
*P*	*<*0.001^*^	*<*0.001^*^	
**Cistern magna volume (cm**^**3**^**)**			
Preoperation	1.34 ± 0.57	1.25 ± 0.55	0.338
Postoperation	3.89 ± 0.96	2.85 ± 0.82	*<*0.001^*^
*P*	*<*0.001^*^	*<*0.001^*^	
**Fourth ventricle volume (cm**^**3**^**)**			
Preoperation	2.01 ± 0.52	1.94 ± 0.52	0.414
Postoperation	1.93 ± 0.49	1.96 ± 0.54	0.733
*P*	0.23	0.722	
Normal rate of cerebellar tonsils [*n*(%)]	50 (73.5%)	47 (52.22%)	0.006^*^
Syrinx remission rate	68.26% ± 26.05%	58.22% ± 36.02%	0.044^*^

^*^p < 0.05.

**Table 4:** Cerebrospinal fluid hydrodynamic parameters.

		PFDRT (*n* = 68)	PFDD (*n* = 90)	*P*
**Peak systolic velocity of CSF (cm/s)**				
Preoperation		6.40 ± 3.07	6.26 ± 3.21	0.772
Postoperation		3.46 ± 1.89	3.74 ± 2.26	0.824
*P*		<0.001^*^	<0.001^*^	-
**Peak diastolic velocity of CSF (cm/s)**				
Preoperation		5.33 ± 3.21	5.79 ± 3.67	0.405
Postoperation		3.08 ± 1.46	3.28 ± 1.83	0.332
*P*		<0.001^*^	<0.001^*^	-
**Net flow of CSF (mL)**				
Preoperation		0.009 ± 0.006	0.010 ± 0.006	0.913
Postoperation		0.120 ± 0.003	0.121 ± 0.003	0.926
*P*		<0.001^*^	<0.001^*^	-

^*^p < 0.05.

### 3.3 Results of postoperative complications

The CCOS score of patients in the PFDRT and PFDD group was 14.00 ± 1.48 vs. 13.14 ± 2.07, respectively (*p* = 0.003). We defined scores of 4–12 as untreated and scores of 13–16 as cured; accordingly, the clinical cure rate of patients in the PFDRT and PFDD group was 85.3% and 64.4%, respectively (*p* = 0.003), indicating that the PFDRT group had a better clinical outcome than the PFDD group. The most common postoperative complication in both groups was aseptic inflammation, with rates of 19.1% and 7.7% in the PFDRT and PFDD group, respectively (*p* = 0.034). There was no significant difference in the rates of other postoperative complications, such as subcutaneous effusion, pseudomeningocele, and secondary surgery, between the two groups (*p* > 0.05). However, the incidence of postoperative complications was higher in the PFDRT group than in the PFDD group (Table 5).

**Table 5:** Postoperative complications.

Feature	PFDRT (*n* = 68)	PFDD (*n* = 90)	*P*
CCOS	14.00 ± 1.48	13.14 ± 2.07	0.003^*^
**Evaluation of clinical outcomes**			
Cure (CCOS 13–16)	58 (85.3%)	58 (64.4%)	0.003^*^
Not cured (CCOS 4–12)	10 (14.7%)	32 (35.3%)	
Postoperative aseptic inflammation	13 (19.1%)	7 (7.7%)	0.034^*^
Subcutaneous effusion	5 (7.4%)	5 (5.6%)	0.974
Pseudomeningocele	2 (2.9%)	1 (1.1%)	0.806
Secondary surgery	2 (2.9%)	1 (1.1%)	0.806

^*^p < 0.05.

### 3.4 Results of prognosis-related factors

Univariate logistic regression analysis affecting the clinical outcomes of the patients showed that different surgical procedures (*p* = 0.004), disease duration (*p* = 0.028), age (*p* = 0.018), preoperative cisterna magna volume (*p* = 0.014), cerebellum-related signs (*p* = 0.022), and systolic CSF peak velocity (*p* = 0.008) significantly affected the clinical outcome. The above variables were subsequently included in the multivariate logistic regression analysis, which revealed that age (*p* = 0.021) and cerebellar-related signs (*p* = 0.044) were independent risk factors for patient outcomes. Additionally, preoperative cisterna magna volume (*p* = 0.043) and systolic CSF peak velocity (*p* = 0.036) were independent factors for clinical outcomes. Furthermore, patients undergoing PFDRT had better clinical outcomes than patients undergoing PFDD (*p* = 0.002; Tables 6, 7).

**Table 6:** Univariate logistic analysis of CCOS.

Parameters	*b*	*SE*	*P*	*OR*	*95%CI*
**Different surgery**					
PFDRT vs. PFDD^#^	1.163	0.407	0.004^*^	3.200	1.441–7.107
**Sex**					
Male vs. female^#^	0.049	0.400	0.093	0.952	0.435–2.085
High blood pressure	0.635	0.374	0.089	1.886	0.907–3.923
Time of surgery (h)	−0.033	0.190	0.862	0.967	0.667–1.404
Time of hospital (day)	−0.055	0.030	0.067	0.947	0.893–1.004
Duration of symptoms (month)	−0.006	0.003	0.028^*^	0.994	0.989–0.999
Preoperation diameter of syrinx (mm)	0.034	0.071	0.626	1.035	0.901–1.189
Preoperation VI	0.381	0.856	0.656	1.463	0.273–7.828
Age (years)	−0.044	0.019	0.018^*^	0.957	0.923–0.993
Preoperation volume of syrinx (cm^3^)	−0.034	0.047	0.464	0.966	0.881–1.059
Preoperation cistern magna volume (cm^3^)	0.944	0.383	0.014^*^	2.571	1.214–5.445
Preoperation fourth ventricle volume (cm^3^)	0.228	0.363	0.529	1.257	0.617–2.561
Preoperation tonsil descent (mm)	−0.019	0.041	0.648	0.981	0.905–1.064
Paraesthesia	−0.204	0.373	0.585	1.226	0.590–2.549
Cough-related headache	0.197	0.395	0.617	0.821	0.379–1.780
Non-cough headache	1.458	1.061	0.169	0.233	0.029–1.861
Motor dysfunction	−0.713	0.381	0.061	2.040	0.968–4.301
Cervical bulbar compression	−0.633	0.931	0.479	0.531	0.086–3.294
Posterior neurological signs	−1.064	0.837	0.204	0.345	0.067–1.781
Cerebellar-related signs	−1.544	0.673	0.022^*^	0.214	0.057–0.802
Preoperation peak systolic velocity of CSF	0.202	0.076	0.008^*^	1.223	1.054–1.421
Preoperation peak diastolic velocity of CSF	0.022	0.053	0.680	1.022	0.921–1.134
Preoperation net flow (mL/s)	1.758	4.108	0.669	5.799	0.002–18.126

^*^p < 0.05; ^#^Reference object.

**Table 7:** Multifactorial logistic analysis of CCOS.

Parameters	*b*	*SE*	*P*	*OR*	*95% CI*
**Different surgery**					
PFDRT vs. PFDD^#^	1.495	0.474	0.002^*^	4.460	1.762–11.288
Duration of signs	−0.002	0.003	0.446	0.998	0.992–1.004
Age	−0.052	0.023	0.021^*^	0.949	0.907–0.992
Preoperation cistern magna volume	0.891	0.440	0.043^*^	2.437	1.030–5.769
Cerebellar-related signs	−1.597	0.795	0.044^*^	0.203	0.043–0.961
Preoperation peak systolic velocity of CSF	0.173	0.082	0.036^*^	1.189	1.011–1.397

^*^p < 0.05; ^#^Reference object.

In this study, the syrinx response rate was used as an indicator to evaluate the imaging outcomes of patients, and the results showed that there were differences based on surgical procedure (r = 0.184, *p* = 0.021), age (r = −0.197, *p* = 0.013), and movement disorders (r = −0.163, *p* = 0.041). In the bivariate analysis, some covariates showed critical statistical variability (*p* < 0.100), such as the preoperative syrinx diameter (r = −0.149, *p* = 0.062). The above variables were included in the multiple linear regression analysis, and the results showed that different surgical methods were positively correlated with the syrinx remission rate (b = 0.119, *p* = 0.014), preoperative syrinx diameter was negatively correlated with the syrinx remission rate (b = −0.022, *p* = 0.018), and age was negatively correlated with the syrinx remission rate (b = −0.007, *p* = 0.002; Tables 8, 9).

**Table 8:** Syrinx remission rate bivariate correlation analysis.

Parameters	*R*	*P*
**Different surgery**		
PFDRT vs. PFDD^#^	0.184	0.021^*^
**Sex**		
Male vs. Female^#^	−0.051	0.528
High blood pressure	−0.043	0.593
Time of surgery (h)	0.014	0.859
Time of hospital (day)	−0.020	0.799
Duration of signs (month)	−0.077	0.334
Preoperation diameter of syrinx (mm)	−0.149	0.062
Preoperation VI	−0.069	0.391
Age (years)	−0.197	0.013^*^
Preoperation volume of syrinx (cm^3^)	0.068	0.394
Preoperation cistern magna volume (cm^3^)	0.014	0.858
Preoperation fourth ventricle volume (cm^3^)	0.002	0.984
Preoperation Tonsil descent (mm)	0.102	0.201
Paraesthesia	0.027	0.736
Cough-related headache	0.029	0.715
Non-cough headache	0.062	0.436
Motor dysfunction	−0.163	0.041^*^
Cervical bulbar compression	0.121	0.131
Posterior neurological signs	0.048	0.549
Cerebellar-related signs	0.002	0.985
Preoperation peak systolic velocity of CSF	0.060	0.456
Preoperation peak diastolic velocity of CSF	0.031	0.697
Preoperation net flow (mL/s)	0.003	0.970

^*^p < 0.05; ^#^Reference object.

**Table 9:** Syrinx remission rate multiple regression analysis.

Parameters	*b*	*t*	*P*	VIF
**Different surgery**				
PFDRT vs. PFDD^#^	0.188	2.476	0.014^*^	1.014
Preoperation diameter of syrinx (mm)	−0.181	−2.381	0.018^*^	1.022
Age (years)	−0.237	−3.109	0.002^*^	1.029
Motor dysfunction	−0.149	−1.968	0.051	1.007

^*^p < 0.05; ^#^Reference object.

## 4 Discussion

Previous studies have shown that early surgical intervention in adult patients with clinical symptoms, especially those complicated with SM, can achieve relatively satisfactory results (21). This long-term follow-up study shows that both PFDRT and PFDD procedures can provide clinical benefits to patients. However, PFDRT promoted a higher SM remission rate, a higher clinical cure rate, and a higher rate of postoperative complications than PFDD. From the perspective of surgical operation, although PFDRT is more invasive than PFDD and can easily cause blood contamination of the subarachnoid space, PFDRT removes the herniated cerebellar tonsils, alleviates nerve compression symptoms, expands the cistern magna, and restores CSF cycle to some extent. This is consistent with the views of Fan et al. (22). Meanwhile, Batzdorf et al. (23) pointed out that cerebellar tonsillectomy can fundamentally solve the problems of cerebellar tonsil compression in the medulla, brainstem, and cerebellum and reduce the possibility of contact between the cerebellar tonsil and the cervical nerve root and further alleviate the symptoms of headache in patients. Some authors recommend cerebellar tonsil resection, whereas others do not recommend it (24, 25). Jia et al. (26) also studied the impact of PFDRT and PFDD on CM-I combined with SM patients, but different results were obtained because the follow-up period was shorter and the prognostic assessment criteria were different from those in this study.

Previous studies have reported that the formation of the syrinx is closely related to abnormal CSF flow dynamics at the craniocervical junction. CSF, which was propelled by herniated cerebellar tonsils, enters the spinal cord through the peripheral spinal vascular and interstitial space at each cardiac cycle, forming syrinx (27). Fluids in the SM produce longitudinal oscillations with the cardiac cycle that promote SM progression. The above-mentioned studies provided a theoretical basis for CM-I combined with SM patient treatment and further supported the effectiveness of PFDRT and PFDD surgery. This study showed that the maximum postoperative diameter of syrinx VI, the subtonsil hernia length, and syrinx volume were significantly reduced (*p* < 0.001), indicating that the effects in both surgical procedures could effectively reduce the syrinx of patients, which is consistent with the results of most studies (22, 28–30). A permanent loss of central spinal cord structures occurs if the obstruction to CSF flow is not relieved (31). Therefore, improving CSF flow at the foramen magnum may reduce the size of the cavity and arrest neurological progression.

Since the early 1990s, multiple studies have assessed CSF flow in CM-I. However, differences in design and varied approaches in the presentation of results and conclusions make it difficult to fully comprehend the role of MRI of CSF flow in CM-I (20). With the continuous progress of MRI technology, researchers used PC-MRI technology to non-invasively measure the characteristics of CSF flow in the patient craniocervical junction. In a cardiac cycle, the maximum peak flow velocity of CSF in most CM-I patients increases, and even flows like a jet; however, the net flow rate of CSF decreases (17). For our study, surgical intervention was associated with positive clinical outcomes when accompanied by improvement in CSF flow as seen on postoperative PC-MRI. The peak systolic velocity of preoperative CSF dynamics was significantly higher than postoperative (*p* < 0.05), and the peak velocity of systole was an independent factor affecting the clinical outcomes of patients. For every 1 cm/s increase in systolic peak velocity, the clinical improvement rate increased by 18.9%. The results of quantitative analysis of CSF in patients preoperative and postoperative showed that the bony bondage and dural compression, which caused cerebellar subtonsil hernia, were removed and improved the CSF circulation pathway; the CSF was recovered to a normal state, the subarachnoid space was unobstructed, the exit of the fourth ventricles was clear, and the SM was significantly reduced. The expansion of the cisterna magna relieves the spinal cord subarachnoid obstruction, increases the effective space of the subarachnoid space, constructs the CSF flow of the cisterna magna and nearby areas, and restores the CSF pressure gradient relief mechanism, thus preventing the progression of the disease. Notably, Ciaramitaro et al. suggested that, at a long-term follow-up, the decrease in the fourth ventricular area after surgery and improved CSF flow in the craniocervical junction has a positive impact on patient prognosis (14). This study analyzed changes in fourth ventricular volume, peak CSF velocity, and net CSF flow, and the results were different from the above research.

CSF hydrodynamic parameters have been increasingly recognized as a predictive factor for the surgical effect and prognosis. Previous studies have revealed that the normalization of CSF dynamic parameters was related to the improvement in clinical symptoms, and patients with greater abnormal preoperative CSF flow had a better clinical response to suboccipital decompression (14, 32–34). Fakhri et al. (15) and Shaffer et al. (35) showed that normalization of CSF dynamic parameters could be seen in MRI of symptomatic CM patients after operation. McGirt et al. (36) used PC-MRI in 130 patients with CM-I to determine the relationship between the change in CSF dynamics and the prognosis of patients. His results showed that the preoperative CSF flow at the craniocervical junction was close to normal, and the postoperative clinical symptoms of patients did not improve significantly. Meanwhile, CM patients with normal preoperative CSF hydrodynamic parameters were 4.8 times more likely to have unrelieved postoperative symptoms than those with abnormal preoperative CSF hydrodynamic parameters, two times more likely to relapse than patients with abnormal CSF hydrodynamics.

This study showed that age was an independent risk factor for prognosis, with older patients having a worse clinical prognosis and a lower remission rate of SM, which was same as previous research studies (14, 37–39). The spinal central tube is the main site of SM expansion in CM-I; the central tube and surrounding areas are the site of extracellular fluid transport and absorption; and mechanical or functional disturbances of these pathways can lead to central canal enlargement and interstitial edema in the surrounding area (40). With aging, the human central tube usually exhibits physiological obstruction, leading to disruption of the central tube absorption mechanism and increasing the likelihood of SM (41). This mechanism seems to help explain the lower void remission rate in older patients. Cerebellar subtonsillar herniation can cause stretching or distortion of cerebellar tissue, which can cause cerebellum-related symptoms (42). This study found that cerebellar-related symptoms were independent risk factors for patient clinical outcomes. Meanwhile, the present study speculated that long-term pulling or distortion of the cerebellum can lead to cerebellar ischemia and necrosis, which can lead to difficult neurological recovery, which further confirms the necessity of early surgery in patients with clinical symptoms. We found in a previous short-term study (38) that dyskinesia was an independent risk factor for patient prognosis. In this long-term study, our conclusions differ from a previous study. We speculate that with the increase of time and individual rehabilitation exercises, the patient’s neurological function can be gradually restored.

## 5 Conclusion

In conclusion, this study suggested that, in a long-term follow-up, although both surgical procedures are effective in treating patients with CM-I and SM, PFDRT is better than PFDD; age and cerebellar-related signs independently affect patient’s prognosis. Additionally, an effective prognosis evaluation index can be developed for patients, based on imaging characteristics, such as preoperative cisterna magna volume, preoperative syrinx diameter, and preoperative CSF hydrodynamic parameters to guide clinical work.

## Data availability statement

The raw data supporting the conclusions of this article will be made available by the authors, without undue reservation.

## Ethics statement

Ethical approval was not required for the study involving humans in accordance with the local legislation and institutional requirements. Written informed consent to participate in this study was not required from the participants or the participants’ legal guardians/next of kin in accordance with the national legislation and the institutional requirements. Written informed consent was obtained from the individual(s) for the publication of any potentially identifiable images or data included in this article.

## Author contributions

YH and MZ contributed to the conception and design of the study. MZ, CD, and MW organized the database. MZ and YH performed the statistical analysis. YH wrote the first draft of the manuscript. YH, DS, and FG wrote sections of the manuscript. All authors contributed to the article and approved the submitted version.
